# ^13^C NMR spectroscopy applications to brain energy metabolism

**DOI:** 10.3389/fnene.2013.00009

**Published:** 2013-12-09

**Authors:** Tiago B. Rodrigues, Julien Valette, Anne-Karine Bouzier-Sore

**Affiliations:** ^1^Cancer Research UK Cambridge Institute and Department of Biochemistry, University of CambridgeCambridge, UK; ^2^Commissariat à l’Energie Atomique, Institut d’Imagerie Biomédicale, Molecular Imaging Research CenterFontenay-Aux-Roses, France; ^3^Centre de Résonance Magnétique des Systèmes Biologiques, UMR 5536, Université Bordeaux Segalen - Centre National de la Recherche ScientifiqueBordeaux, France

**Keywords:** ^13^C NMR spectroscopy, brain metabolism, neuron, astrocyte, neuroglial coupling, metabolic modeling, hyperpolarized NMR

## Abstract

^13^C nuclear magnetic resonance (NMR) spectroscopy is the method of choice for studying brain metabolism. Indeed, the most convincing data obtained to decipher metabolic exchanges between neurons and astrocytes have been obtained using this technique, thus illustrating its power. It may be difficult for non-specialists, however, to grasp thefull implication of data presented in articles written by spectroscopists. The aim of the review is, therefore, to provide a fundamental understanding of this topic to facilitate the non-specialists in their reading of this literature. In the first part of this review, we present the metabolic fate of ^13^C-labeled substrates in the brain in a detailed way, including an overview of some general neurochemical principles. We also address and compare the various spectroscopic strategies that can be used to study brain metabolism. Then, we provide an overview of the ^13^C NMR experiments performed to analyze both intracellular and intercellular metabolic fluxes. More particularly, the role of lactate as a potential energy substrate for neurons is discussed in the light of ^13^C NMR data. Finally, new perspectives and applications offered by ^13^C hyperpolarization are described.

## INTRODUCTION

The brain is metabolically the most energy-consuming organ. Adequate brain physiology depends on the unceasing supply of proper amounts of oxygen and plasma glucose (Glc). Consequently, limitations in the delivery of these two cerebral substrates cause most physiopathological states (Nicholls, 2007; Okada and Lipton, 2007).

Classical approaches to study cerebral metabolism, both in physiological and in physiopathological conditions, required the use of optical methods or radioactive isotopes and the isolation and purification of the enzymes or transport systems involved to study the corresponding *in vitro* kinetics (Bachelard, 1989; Clark and Lai, 1989; Sokoloff, 1989). This reductionist approach provided essential information on the operation of the central nervous system (CNS), despite the limitations brought by the small amounts of the involved proteins present in cerebral tissues and by the fact that its utilization was circumscribed to *postmortem* biopsies or cerebral extracts.

The remarkable advance during the past decades of powerful tools for investigating the human brain has had a tremendous impact on our ability to investigate and understand brain function. Autoradiography and positron emission tomography (PET) methods have been developed based on the measurement of regional Glc consumption, after the administration of 2-deoxyglucose, either labeled with ^14^C or with ^18^F, respectively (Sokoloff, 1981; Wienhard, 2002; Herholz and Heiss, 2004). These methodologies can be used to determine the regional accumulation of 2-(^14^C or ^18^F)-deoxyglucose-6-phosphate, virtually unmetabolizable analogs of glucose-6-phosphate, using autoradiography or PET. Autoradiography provides *ex vivo* images of the regional accumulation of radioactive 2-deoxyglucose-6-phosphate (or other radioactive substrates such as acetate and butyrate, among others), as reflected in photographic plates obtained from sections of fixed brain tissue. PET produces *in vivo*, possibly dynamic, images of the regional uptake of 2-(^18^F)-deoxyglucose (FDG, or other positron emitters) in different brain sections, as resolved tomographically by a coronal arrangement of positron selective gamma cameras. Both approaches allow the determination of cerebral metabolic rates for Glc transport and phosphorylation (CMR_glc_) in different cerebral regions after appropriate modeling of the underlying tracer kinetics (Price, 2003). However, these radioactive approaches are limited in resolution and chemical specificity, making it not possible to investigate the downstream metabolism of Glc after the first glycolytic enzymatic step. Similarly, functional magnetic resonance imaging (fMRI) indirectly allowed the investigation of the hemodynamic and blood oxygenation changes associated with sensory or motor stimulation (Heeger and Ress, 2002). Despite their importance, FDG uptake or fMRI provided no information on the pathways and metabolic interactions underlying the cerebral activation process. This implies that further advances in this area would involve necessarily the use of additional methodologies. From this perspective, genome cloning and sequencing techniques, as well as the important development of novel nuclear magnetic resonance (NMR) approaches have overcome many of the limitations of the traditional strategies, as explained below. In particular, sequencing of the human and mouse genomes has provided a broad understanding of the different isoforms of enzymes and transporters present in the brain, without the need to isolate and purify the corresponding proteins (International Human Genome Sequencing Consortium, 2001; Mouse Genome Sequencing Consortium, 2002). These genomic methods, however, do not allow the investigation of the function and *in vivo* performance of the genes sequenced or cloned. It is in this respect that NMR technologies have become more helpful, providing the quantitative assessment of transport steps, metabolic fluxes and cellular compartmentalization of glycolysis, pyruvate (Pyr) oxidation, and tricarboxylic acid (TCA) cycle, among other pathways, in a plethora of neural systems ranging from primary cell cultures to the intact rodent or human brain (Gruetter etal., 2003; Shulman etal., 2004; Rodrigues and Cerdán, 2005).

Pioneering NMR approaches to cerebral energetics begun with the application of ^31^P NMR (Moore etal., 1999). These ^31^P NMR spectra from rodent, cat, dog, or human brain – depicted resonances from adenosine triphosphate (ATP), phosphocreatine (PCr), inorganic phosphate (Pi), phosphomonoesters (PME, mainly phosphorylethanolamine), and phosphodiesters (PDE, glycerophosphorylcholine; Hilberman etal., 1984; Komatsumoto etal., 1987; Nioka etal., 1991). With this technique it was possible to follow non-invasively the rates of PCr breakdown and recovery after hypoxic and ischemic episodes.

Nowadays, the most extended NMR approach to explore brain in the clinic is ^1^H NMR spectroscopy (Burtscher and Holtas, 2001). ^1^H NMR spectra from human or rodent brain show resonances from the methyl group of *N*-acetyl-aspartic acid (NAA), the methyl groups of creatine (Cr) and PCr, the trimethylammonium groups of choline (Cho) containing compounds and the *myo*-inositol (Ins), glutamate (Glu), glutamine (Gln), and γ-aminobutyric acid (GABA) resonances, among others. Ins and NAA are thought to represent the glial and neuronal contributions to the observed voxel, respectively. Remarkably, lactate (Lac) becomes evidently observable under hypoxic or ischemic conditions, providing a proof of augmented net glycolytic flux under these conditions. However, ^1^H NMR spectroscopy has the limitation of poor signal dispersion, compared to other commonly used spin nuclei, with the consequent severe overlap problems.

^13^C NMR approaches constitute probably the most elaborated, chemically specific, tool to follow the metabolic fate of ^13^C-labeled substrates in the brain, both *in vivo* and *in vitro* (de Graaf etal., 2003b; Gruetter etal., 2003; Garcia-Espinosa etal., 2004; Rodrigues etal., 2009). Since the first ^13^C NMR spectroscopy study of a living organism, describing the metabolism of [1-^13^C]Glc by an eukaryotic cell system (Eakin etal., 1972), this approach developed into a powerful method for metabolic research with cells, perfused organs, *in vivo* animals and humans (Morris and Bachelard, 2003). It enabled measuring metabolic processes as they occur in their intracellular environment. Furthermore, it continues to provide unique information, not accessible from previously used approaches.

^13^C NMR spectroscopy allows detecting resonances from ^1^^3^C, the only stable isotope of carbon having a magnetic moment. The natural abundance (NA) for ^13^C is approximately 1.1% of the total carbon and its magnetogyric ratio is approximately one-fourth of that of the proton. These two circumstances make ^13^C NMR spectroscopy a relatively insensitive technique (Friebolin, 1991). The sensitivity can be improved noticeably by using ^13^C-enriched substrates. The combination of ^13^C NMR spectroscopy detection and substrates selectively enriched in ^13^C in specific carbon positions has made it possible to follow *in vitro* and *in vivo* the activity of a large variety of metabolic pathways. These include glycolysis and the pentose phosphate pathway, glycogen synthesis and degradation, gluconeogenesis, the TCA cycle, ketogenesis, ureogenesis, and the Glu–Gln/GABA cycle in brain, among others (Cerdan and Seelig, 1990; Kunnecke, 1995; Morris and Bachelard, 2003; Rodrigues etal., 2007). The ^13^C NMR approach also enables to investigate the activities of the neuronal and glial TCA cycles *invitro* and *in vivo*, providing direct insight into cerebral metabolic compartmentalization (Cerdan etal., 2009).

The design of ^13^C NMR experiments with selectively ^13^C-enriched substrates is similar to the classical radiolabeling experiments using ^14^C. An important difference is that ^13^C precursors are administered in substrate amounts, while ^14^C substrates are used in tracer amounts. Despite this, ^13^C NMR presents important advantages over methodologies using ^14^C: (i) the metabolism of the ^13^C-labeled substrate can be followed in real-time, *insitu* and non-invasively (Szyperski, 1998; Morris and Bachelard, 2003); (ii) even if tissue extracts are prepared, the detection of ^13^C in the different carbon resonances of a specific metabolite does not require separation and carbon by carbon degradation, a prerequisite in the experiments with radioactive ^14^C (Dobbins and Malloy, 2003); and (iii) when two or more ^13^C atoms occupy contiguous positions *in the same metabolite molecule* it will give rise to isotope effects, called homonuclear spin-coupling, that lead to the appearance of multiplets (instead of single resonances). The analysis by ^13^C NMR of these homonuclear spin-coupling patterns represents an enormous gain in the information obtained as compared to the classical radioactive ^14^C experiments (Dobbins and Malloy, 2003). As a counterpart to these advantages, ^13^C NMR is significantly less sensitive than other conventional metabolic techniques like radioactive counting, mass spectrometry, and fluorimetric or spectrophotometric methods.

Investigation of metabolic pathways using ^13^C NMR spectroscopy is comprised of three main tasks: (i) the infusion of a ^13^C-labeled substrate; (ii) the detection of ^13^C-labeled metabolites following substrate consumption; and (iii) the metabolic modeling of measured ^13^C enrichments to quantitatively derive metabolic fluxes. In general, these three tasks are closely interconnected. Each of them imposes constraints on the two others, and all three must be designed depending on metabolic pathways that are to be investigated. The choice of the substrate (such as Glc, acetate, Pyr, among others) will allow more or less specific feeding of a specific cell type (such as neurons and astrocytes). This will, in-turn, impose the choice of modeling for these cells, and may drive the NMR methodological choices to measure ^13^C labeling for cell-specific metabolites (such as Glu, Gln, GABA, among others). Alternatively, the ability of ^13^C spectroscopy methods to resolve certain peaks on NMR spectra may lead to the refinement of metabolic models, while the inability to resolve peaks may impose the choice of a labeled substrate whose consumption does not lead to the formation of species with spectral overlap.

Due to the connection between biological questions and methodological issues, a good understanding of the practical implementation of ^13^C experiments, with associated caveats and pitfalls, is a prerequisite to any investigation and discussion of metabolism based on ^13^C studies. In this review, we will initially provide a simple picture of brain energy metabolism, with a level of details commensurable with NMR accessible information, and explain how ^13^C nuclei from different substrates flow through metabolic pathways. Then, spectroscopic acquisition techniques will be reviewed, with associated advantages, drawbacks, and technical difficulties. The basis of metabolic modeling to derive quantitative flux values will be then explained. Finally, we will address two different models of neuroglial coupling : the astrocyte–neuron lactate shuttle (ANLS) model (Pellerin and Magistretti, 1994; Pellerin etal., 2007) and the redox switch/redox coupling hypothesis (Cerdan etal., 2006; Ramirez etal., 2007).

## THE JOURNEY OF CARBON: METABOLIC FATES OF LABELED SUBSTRATES

### FUELS FOR THE BRAIN

**FIGURE 1:**
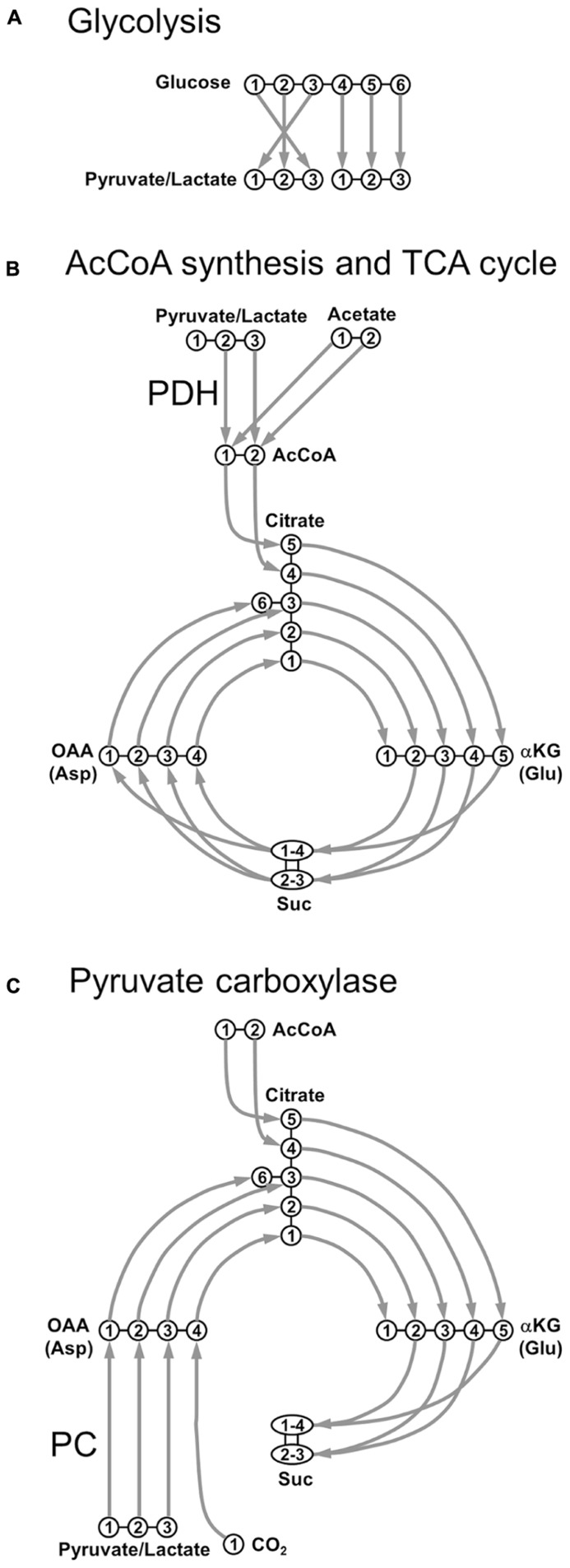
**Transfer of individual** ^**13**^C nuclei during the main steps of oxidative metabolism. **(A)** Glycolysis; **(B)** acetyl-CoA synthesis through pyruvate dehydrogenase (PDH) and TCA cycle; **(C)** pyruvate carboxylase (PC). AcCoA, acetyl-CoA; αKG, α-ketoglutarate; Asp, aspartate; Glu, glutamate; OAA, oxaloacetate; Suc, succinate.

The quasi-universal energy molecule of living systems is ATP, which is predominantly synthesized during aerobic cellular respiration (Gjedde, 2007). A central mechanism of aerobic cellular respiration is the TCA cycle, where fuel molecules undergo complete oxidation, ultimately leading to ATP synthesis through oxidative phosphorylation in mitochondrial cristae. When these fuel molecules are labeled with ^13^C and continuously infused, their degradation in the TCA cycle will lead to the progressive incorporation of ^13^C into metabolic intermediates and by-products (Rodrigues and Cerdán, 2007). The journey of ^13^C nuclei is summarized in **Figure 1**.

Under physiological conditions, the main cerebral substrate is Glc. After crossing the blood–brain barrier (BBB), a Glc molecule originates two Pyr molecules through glycolysis (**Figure 1A**). Pyr can be reduced to Lac by the lactate dehydrogenase (LDH, fast exchange) with the following reversible reaction:

(1) $$Pyruvate\,\;+\;NADH\,\;+\;H^{+}\,\;↔\;lactate\,\;+\;{NAD}^{+}$$

Lactate dehydrogenase is a tetramer composed of different combinations of two subunits, H (isolated from heart) and M (from muscle): H4 (LDH1), H3M (LDH2), H2M2 (LDH3), HM3 (LDH4), and M4 (LDH5). LDH1 is mostly neuronal and its kinetic properties promote the formation of Pyr (Bittar etal., 1996). Conversely, LDH5 is primarily astrocytic and its kinetic characteristics favor mainly Lac formation. Pyr is also transported into mitochondria and decarboxylated to acetyl-CoA (AcCoA) *via* the oxidative pathway (pyruvate dehydrogenase, PDH), as shown in **Figure 1B**. AcCoA enters TCA cycle by irreversibly condensating with oxaloacetate (OAA) to form citrate, which is subsequently converted to α-ketoglutarate (αKG) via isocitrate. αKG is then degraded into succinate (Suc) via succinyl-CoA, where scrambling occurs between C1 and C4 positions, and between C2 and C3 positions, due to the symmetry of the Suc molecule. Suc is then oxidized to fumarate, with flavin adenine dinucleotide (FADH_2_) used as the hydrogen acceptor. The next step is the hydration of fumarate to form malate, and the cycle becoming complete with the oxidation of malate to OAA (**Figure 1B**). Pyr, or even Lac, can be directly supplied to the brain as fuels for TCA cycle. An alternative fuel is acetate, which can be directly converted to AcCoA. This was primarily suggested to happen in astrocytes (Waniewski and Martin, 1998). It was proposed that the main reason acetate is a relatively poor substrate for neurons was due to transporter affinity. This study was based on poor uptake of acetate by synaptosomal fractions compared to astrocytes, not measuring the uptake of acetate by neurons in this work. Further studies revealed that metabolism of acetate is tightly controlled at the enzyme level, via changes in the acetylation status of AcCoA and is not regulated by restriction of uptake (Rae etal., 2012).

In addition to PDH, Pyr may also enter the TCA cycle via the anaplerotic pathway, after its carboxylation to OAA, through the pyruvate carboxylase (PC), as depicted in **Figure 1C**. In contrast to PDH metabolism, which preserves the source of carbon skeletons in TCA cycle, OAA is synthesized *de novo* by PC. The total number of carbon skeletons in the TCA cycle is therefore increased, consequently requiring a net efflux before a turn has been completed. This anaplerotic pathway is mainly glial, due to the specific astrocytic localization of PC (Shank etal., 1985; Sonnewald and Rae, 2010).

### LABELING OF NMR-VISIBLE AMINO ACIDS

Nuclear magnetic resonance detection threshold is typically in the millimolar (mM) range, which is above the typical concentration of most TCA cycle intermediates, including αKG and OAA. However, these intermediates are in fast exchange with amino acids, which exist in concentrations that are above the detection threshold, making ^13^C-labeling measurements possible. In particular, αKG is in fast exchange with Glu through aspartate aminotransferase, with identical labeling patterns for the keto acid and the amino acid pools. In neurons, neuronal OAA (OAA_n_) is in exchange with a pool of Asp, with identical labeling patterns as well.

Exploring **Figure 1**, it is relatively easy to track ^13^C labeling along metabolic pathways from labeled substrates to amino acids. For example, following [1-^13^C]Glc or [1,6-^13^C_2_]Glc infusions, generation of [3-^13^C]pyruvate is observed, which via PDH leads to the labeling of αKG and Glu at the C4 position during the first turn of the TCA cycle. During the second turn of the TCA cycle, ^13^C label is then transferred to Glu C2 and Glu C3. Note that comparing to [1,6-^13^C_2_]Glc, the use of [1-^13^C]Glc as precursor will lead to a 50%-dilution in the ^13^C enrichment of Pyr at the end of the glycolysis.

### NEUROTRANSMISSION AND METABOLIC INTERACTIONS BETWEEN NEURONS AND ASTROCYTES

**FIGURE 2:**
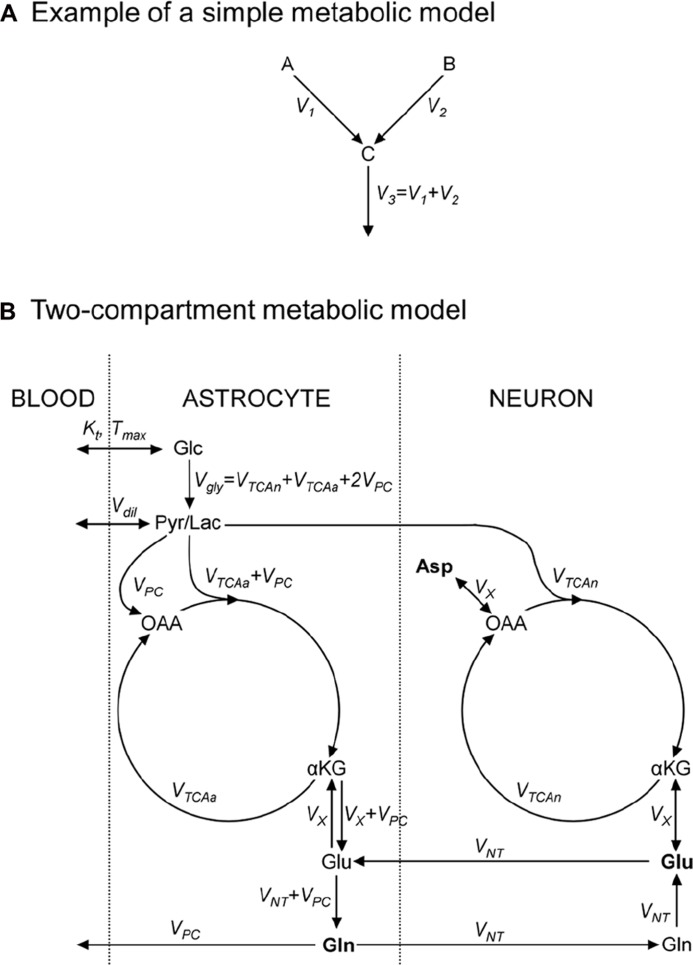
**Metabolic models consist in an ensemble of metabolic pools, connected by metabolic fluxes respecting the steady-state condition for metabolic pools.(A)** Example of a simple metabolic model used to illustrate how to write ^13^C-labeling equations; metabolic pools A and B yield C at rates *V*_1_ and *V*_2_, respectively; **(B)** a “classical” two-compartment metabolic model (see main text for model’s description). αKG, α-ketoglutarate; Asp, aspartate; Glc, glucose; Gln, glutamine; Glu, glutamate; Lac, lactate; OAA, oxaloacetate; Pyr, pyruvate; Suc, succinate.

The TCA cycle plays a central role in brain metabolism because sugars, fatty acids, and amino acids are oxidized in this pathway. This metabolic route provides numerous intermediates for cerebral biosynthetic pathways, including the neurotransmitters Glu and GABA. In the case of brain cell compartmentalization, two different TCA cycles may be considered as functioning in parallel: an astrocytic and a neuronal cycle. The existence of these two cycles, with different kinetics, was firstly demonstrated in the 1960s. When ^14^C-labeled Glu was administered to rats, brain radioactivity was mainly found in the form of Gln (Berl etal., 1961), showing that there is an exchange between Glu and Gln. In addition, the specific activity recovered was higher for Gln than for Glu. Therefore, a pool of Glu exists in the brain that is not exchanged. The use of ^15^N-labeled ammonium confirmed the existence of two pools of Glu (Berl etal., 1962), originating the concept that there are two different Glu compartments: a “small” compartment where the Glu–Gln conversion is fast, and a “large” compartment in which the renewal of Glu is much slower. Subsequently, it was shown that glutamine synthetase (GS), an enzyme responsible for the synthesis of Gln, was found mainly in astrocytes (Norenberg and Martinez-Hernandez, 1979). On the other hand, the enzyme responsible for its conversion to Glu, glutaminase, was found mainly in the neurons (Patel etal., 1982). Thereafter, the two compartments were assigned to the two cell types: neurons representing the “large” compartment and astrocytes the “small” one. One of the main consequences of the metabolic compartmentation concept is that there is a Glu–Gln cycle between neurons and astrocytes (cf. **Figure 2B**). In this intercellular metabolic exchange, Gln is synthesized by astrocytes and then transferred to the neuronal compartment, where it is converted to Glu. The Glu, a major neuronal signaling molecule, is released by the neurons into the synaptic cleft during neuronal activity, and then taken up by astrocytes, where it is transformed back into Gln (Badar-Goffer etal., 1990; Cerdan etal., 1990; Shank etal., 1993; Lapidot and Gopher, 1994). This exchange has been widely studied (Schousboe and Hertz, 1981; Waniewski and Martin, 1986; Erecinska and Silver, 1990), including *in vivo* using ^13^C NMR spectroscopy (for review, see Rothman etal., 2011), and various Glu transporters have been found on astrocytes (Erecinska and Silver, 1990; Flott and Seifert, 1991; Danbolt, 2001; Huang and Bergles, 2004).

The metabolic relationship between neurons and astrocytes appears, however, to be much more complex than the existence of a simple Glu–Gln cycle. Indeed, it is possible to show that the uptake of Gln by neurons does not offset the continuous loss of Glu (Hertz, 1979). Therefore, the neurons must use other metabolites that are precursors of the synthesis of Glu, as the components of the TCA cycle, and must therefore be equipped with an enzymatic route to allow their net synthesis from Glc. As briefly addressed above, this synthesis occurs mainly through the PC activity (Voet and Voet, 1990). However, it turns out that this enzymatic activity is present only in astrocytes (Yu etal., 1983; Shank etal., 1985), imposing a necessary anaplerotic transfer of carbons from astrocytes to neurons to replenish the neuronal pools of oxidized TCA intermediates.

In GABAergic neurons, Glu is converted to GABA by glutamate decarboxylase, which is subsequently released in the synaptic cleft. Most GABA molecules are recaptured by neurons, but a small fraction is also recaptured by astrocytes and incorporated into the glial TCA cycle.

Additional pathways exist that may impact the ^13^C labeling of NMR-visible metabolites such as Pyr recycling, the transfer of Lac from astrocytes to neurons and the alanine–lactate shuttle between neurons and astrocytes (Waagepetersen etal., 2000; Zwingmann and Leibfritz, 2003).

## ^**13**^C NMR SPECTROSCOPY ACQUISITION TECHNIQUES

The ability to detect ^13^C enrichment in brain amino acids is governed by two parameters: sensitivity and spectral resolution. High signal-to-noise ratio (SNR) means that metabolites can potentially be quantified with lower concentrations or enrichments, in smaller volumes, or in shorter periods. Good spectral resolution means that more resonances (corresponding to more metabolites or more specific positions) can be individually quantified, resulting in a higher chemical specificity. SNR and spectral resolution increase linearly with the magnetic field, although technical challenges become significant *in vivo* (shorter *T*_2_ relaxation times, increased energy deposition in tissues, higher demand on radiofrequency (RF) pulse bandwidth, and poor homogeneity of the RF field). Essentially, two main approaches can be distinguished for ^13^C detection, each trading one of these parameters against the others: direct ^13^C detection and indirect ^13^C detection.

### DIRECT ^13^C DETECTION: CHEMICAL SPECIFICITY

^13^C NMR resonances of brain metabolites span a very broad chemical shift range (~250ppm), in which conveys the ability to resolve virtually all carbon positions in the detectable metabolites. In particular, direct ^13^C spectroscopy allows simultaneously resolving Glu and Gln at C2, C3, and C4 positions, as well as Asp and GABA at positions C2 and C3, even *in vivo* (Gruetter etal., 2003; Henry etal., 2003a). The carboxylic carbons are, in all cases, more difficult to observe because of their long *T*_1_s and significant saturation effects.

Beyond the information about positional enrichment, direct ^13^C spectroscopy allows quantifying isotopomers (i.e., individual molecules labeled at different atomic positions), since it is sensitive to a constant value – called scalar *J* coupling – that is different for each ^13^C neighborhood type. Indeed, the scalar *J* coupling will result in the splitting of singlet resonances, corresponding to a given enriched position, into multiplets for ^13^C nuclei coupled with neighboring ^13^C. This additional information about isotopomers allows one to resolve the activity of different metabolic pathways, as discussed below.

One-bond heteronuclear coupling between ^1^^3^C and ^1^H may compromise spectral resolution and SNR, since it results in the splitting of ^13^C resonances in doublets or multiplets (*J*~130Hz), with reduced peak heights. Therefore, it is generally desirable to perform heteronuclear decoupling during ^13^C acquisition. This is achieved by the application of a RF train at ^1^H frequency, resulting in the effective suppression of the effects of ^1^H–^13^C coupling on ^13^C spectra. Besides technical difficulties associated with the necessity to control two RF chains and to prevent noise injection from the ^1^H transmission chain into the ^13^C acquisition chain, decoupling may become problematic for *in vivo* application at high field due to the large power deposition in tissues (de Graaf, 2005). It has, however, been shown that detection without decoupling could be achieved in the human brain at 9.4T with acceptable accuracy (concentration uncertainty was 35–90% higher; Deelchand etal., 2006).

The main disadvantage of direct ^13^C spectroscopy is its low sensitivity, derived from the low gyromagnetic ratio of ^13^C. Three different strategies, namely nuclear Overhauser effect (nOe), pulsed polarization transfer (PPT), and cross-polarization (CP; Ernst etal., 1987) have been proposed to transfer polarization (or magnetization) from neighboring ^1^H to ^13^C, both in liquids and *in vivo*. This implies an increase in the ^13^C polarization, ultimately resulting in higher SNR. Like heteronuclear decoupling, these strategies require two transmission channels at ^1^H and ^13^C frequencies.

Nuclear Overhauser effect relies on direct (through-space) dipolar coupling between spins, and refers to the transfer of polarization from ^1^H to ^13^C due to cross-relaxation. This is achieved when RF irradiation is performed at the ^1^H frequency while longitudinal relaxation occurs, which drives the ^13^C thermodynamic equilibrium polarization to a higher value. Assuming that ^13^C relaxation is entirely due to dipolar interaction with ^1^H, nOe increases ^13^C polarization up to a factor 1+0.5×γ_I_/γ_S_ = 3, where γ_I_ and γ_S_ are ^1^H and ^13^C gyromagnetic ratios. Excitation is thus performed both in the ^1^H and ^13^C frequencies, while detection is obtained only in the ^13^C channel.

Cross-polarization and PPT rely on indirect (through-bond) scalar coupling between spins (*J*-coupling), the excitation being initially performed for ^1^H. Then, under the combined effect of *J*-coupling and RF perturbation, polarization is driven to an observable ^13^C state with amplitude corresponding to γ_I_ instead of γ_S_, as would result from direct ^13^C excitation. Ideal CPT and PPT therefore yield up to a γ_I_γ_S_ = 4-fold gain in SNR. For CPT, this is optimally achieved after RF irradiation of ^1^H and ^13^C frequencies during a 1/*J* delay, when the Hartmann–Hahn condition is met (γ_I_ *B*_1__I_ = γ_S_ *B*_1__S_; Hartmann and Hahn, 1962) and high B_1_ amplitudes are used. On the other hand, PPT only requires short RF perturbations (simultaneous 90° pulse at both frequencies at time 1/2*J* after initial excitation). It is therefore particularly interesting for *in vivo* applications due to the limited power deposition, while CPT can yield slightly larger SNR gains. An important feature for the *in vivo* application is that the localization can be fully achieved at the ^1^H frequency before transferring polarization, resulting in better localization accuracy compared to the direct ^13^C localization, due to the narrower ^1^H chemical shift range.

In practice, gains in SNR are significantly smaller than predicted under ideal conditions and vary between different resonances, complicating the quantification process. SNR gains up to 3.5 have been reported in the human brain at 3T, combining nOe and CPT (Klomp etal., 2006).

### INDIRECT ^13^C DETECTION: HIGH SENSITIVITY

As an alternative to detecting ^13^C signal directly, an efficient way to increase these measurements sensitivity is to detect ^1^H bound to ^13^C. SNR gains result mostly from the increased signal voltage, which is proportional to the higher ^1^H thermal equilibrium magnetization – by a factor (γ_I_/γ_S_)^2^ – and to the higher precession frequency – by a factor γ_I_/γ_S_ – compared to ^13^C. At the same time, state-of-the-art coils yield noise voltage increasing roughly linearly with the frequency, i.e., as γ_I_/γ_S_. Therefore, a (γ_I_/γ_S_)^2^~16-fold increase in SNR can be expected when going from direct detection (without polarization transfer) to indirect detection.

Indirect detection is usually based on a proton-observed carbon-edited (POCE) strategy, requiring two transmission channels at ^1^H and ^13^C frequencies. The strategy is based on a standard ^1^H spectroscopy sequence with an additional 180° pulse at ^13^C frequency, being ON or OFF every other scan (Rothman etal., 1985). When the ^13^C pulse is ON, satellite resonances due to coupling between ^1^H and ^13^C nuclei are of opposite sign when compared with the OFF case, while resonances corresponding to ^1^H bound to ^12^C nuclei are unaffected. Therefore, subtracting odd from even scans will result in the cancellation of signal from ^1^H bound to ^12^C, while signal from ^1^H bound to ^13^C will build up.

Heteronuclear decoupling is generally performed by the application of a RF train at ^13^C frequency during the ^1^H acquisition. This is complicated by the large chemical shift range of ^13^C, which imposes a requirement for ultra-broadband decoupling (resulting in high-power deposition) if all resonances on the ^1^H spectra have to be decoupled. Decoupling is performed to increase SNR but also to improve spectral resolution, which is critical when observing ^1^H resonances. Indeed, the ^1^H chemical shift range only spans ~3ppm for the aliphatic portion which covers the metabolites’ resonances of interest. It is generally accepted that resolution of Glu and Gln C4 becomes possible only for B_0_>3T, while resolving Glu and Gln C3 remains problematic even at much higher field (Pfeuffer etal., 1999). Indirect detection of GABA and Asp labeling remains problematic and has only been reported at B_0_ = 7T or above in the rodent brain (Pfeuffer etal., 1999; de Graaf etal., 2003a; Yang etal., 2005; van Eijsden etal., 2010). Therefore, the loss of chemical specificity associated with indirect detection is acceptable mostly for *in vivo* applications where sensitivity is critical, especially when performing a dynamic measurement: collecting multiple spectra during ^13^C-labeled substrate infusion. Indirect ^13^C spectroscopy *in vivo* was extensively reviewed by de Graaf etal. (2003b).

An alternative method has been recently proposed for *in vivo* applications, which presents the unique characteristic of requiring no ^13^C RF pulse-chain. The method is based on the subtraction of ^1^H spectra collected during the ^13^C infusion from a baseline spectra acquired prior to infusion (Boumezbeur etal., 2004). Using this approach, C4 and C3 positions could be resolved for the total “Glu+Gln” pool at 3T. Note that the technique demands extremely stable acquisition (including shimming and coil sensitivity) over the entire experiment.

### NOTE ON SPECTRAL QUANTIFICATION

Analysis of ^13^C spectra has long been performed by simple peak integration, which is possible due to the limited spectral overlap on direct ^13^C spectra. More recently, spectral quantification based on prior knowledge has been introduced, using for example the LCModel software (Provencher, 1993). In this approach, individual spectra of labeled molecules (obtained by experimental measurement or numerical simulation) are linearly combined to fit experimental data. This allows accurate quantification despite partial overlap, which becomes particularly interesting to discriminate different isotopomers around a given resonance, being possible to perform it even *in vivo*, where lines are broader (Henry etal., 2003a). Although still uncommon in direct ^13^C spectroscopy, prior knowledge spectral fitting is now routinely implemented in indirect ^13^C spectroscopy, due to unavoidable overlap on ^1^H spectra.

Absolute quantification, i.e., the determination of metabolite concentration and enrichment (in mM and %^13^C), as required for dynamic metabolic modeling (see below), is generally easier using indirect spectroscopy, due to the presence of internal references of known concentration, such as unlabeled Cr or water. With direct spectroscopy, absolute quantification can be complicated by the different polarization transfer efficiency for the different resonances, and for *in vivo* experiments by the absence of a suitable internal ^13^C reference of known concentration.

## METABOLIC MODELING

Examination of ^13^C enrichment can yield qualitative information about metabolite compartmentalization and the existence and relative importance of metabolic pathways. When seeking quantitative information, one must turn to metabolic modeling, whose basic principle is to mathematically express ^13^C labeling of detected metabolites as a function of the metabolic fluxes underlying the labeling process.

### WRITING EQUATIONS: MASS CONSERVATION AND LABEL INCORPORATION

As an exercise, we should consider two metabolite pools, A and B, yielding a third pool, C, at rates *V*_1_ and *V*_2_ (in μmol/g/min), respectively, and C being then consumed at rate *V*_3_ (**Figure 2A**). A usual assumption is that the size of pool C remains constant:

(2) $$\frac{d\,\left[C\right]}{d\,t}=V_{1}+V_{2}-V_{3}=0\,$$

This imposes that the total influx in the pool is equal to the total efflux from the pool, *V*_3_ = *V*_1_+*V*_2_. We should also assume that ^13^C nuclei, at position *i* in A and *j* in B, both enter the C pool at position *k*. We use A_i_*, B_j_*, and C_k_* to denote molecules labeled at these positions. ^13^C mass conservation imposes that the increase in the C_k_* pool size is equal to the amount of ^13^C entering the pool *minus* what exits the pool at each instant:

(3) $$\frac{d\left[C_{k}^{*}\right]}{dt}=V_{1}\frac{\left[A_{i}^{*}\right]}{\left[A\right]}+V_{2}\frac{B_{j}^{\;*}}{\left[B\right]}-\left(V_{1}+V_{2}\right)\frac{\left[C_{k}^{\;*}\right]}{\left[C\right]}\,$$

A metabolic model typically consists in several equations of the previous type, describing label transfer from infused substrates to metabolic intermediates and, ultimately, to detected metabolites. To favor an efficient solution, the number of differential equations describing the model should be minimized. Equations describing low-concentration intermediates can generally be omitted since their enrichment mimics that of the immediately preceding high-concentration metabolite. Except at steady-state, these systems of differential equations can generally not be solved analytically and require numerical computing to determine what flux values yield the best fit to experimental data.

### TEMPORAL RESOLUTION: STEADY-STATE *VERSUS* DYNAMIC MODELING

To illustrate the impact of temporal resolution on a model, we can assume constant, but different, fractional enrichments for A and B ([A_i_*]/[A] = FE_A_, [B_j_*]/[B] = FE_B_). A common procedure in acquiring these data is to wait a period of time after the start of the ^13^C infusion, ensuring that isotopic steady-state has been reached for [C_k_*]. In this case, Eq. 2 immediately yields, with FE_C_ = [C_k_*](*t* = ∞/[C]:

(4) $$\frac{V_{1}}{V_{2}}=\frac{{FE}_{C}-{FE}_{B}}{{FE}_{A}-{FE}_{C}}\,$$

The ratio *V*_1_ to *V*_2_ can therefore be determined from known values of FE_A_, FE_B_, and FE_C_. In general, metabolic models at steady-state only yield flux ratios, not absolute values.

In contrast, we can also explore how dynamic modeling (i.e., using data collected at different time points) carries richer information. We solve Eq. 2 assuming fractional enrichment (also called specific enrichment) for A and B going instantaneously from ^1^^3^C NA = 1.1% to FE_A_ and FE_B_ at *t*>0:

(5) $$\frac{\left[C_{k}^{\;\;*}\right]\left(t\right)}{\left[C\right]}=\frac{V_{1}FE_{A}\,\;+\;V_{2}FE_{B}}{V_{1}\,\;+\;V_{2}}\,\;+\;\left(NA-\frac{V_{1}FE_{A}\,\;+\;V_{2}FE_{B}}{V_{1}\,\text{ + }V_{2}}\right)e^{-\frac{V_{1}+V_{2}}{\left[C\right]}t}\,\;$$

It appears that the enrichment curve will again carry information about the ratio *V*_1_/*V*_2_ from long-time enrichment, and independently *V*_1_+*V*_2_ from the exponential rise (provided [C] is known). This means that the absolute values of *V*_1_ and *V*_2_ (in μmol/g/min) can now be determined. The ability to assess absolute flux values and, potentially, for a number of fluxes greater than the number of equations is a unique feature of dynamic modeling. However, absolute quantification of concentrations is required.

### FEEDING DYNAMIC MODELS: SUBSTRATE ENTRY INTO THE BRAIN

Dynamic metabolic modeling is complicated by the need to estimate the temporal evolution of substrate’s intracellular concentration and the enrichment as an entry function. Since, in general, these parameters cannot be directly measured, they are calculated from plasma concentrations and enrichments by modeling transport through the BBB. Transport of Glc and monocarboxylic acids through the BBB is a bidirectional process and is best modeled by reversible Michaelis–Menten transport equations (Simpson etal., 2007). Kinetic parameters have been estimated in the mammalian brain for Glc (Gruetter etal., 1998), acetate (Deelchand etal., 2009), and Lac (Boumezbeur etal., 2010). Blood sampling throughout the infusion is required to determine plasmatic concentration and enrichment of the investigated substrate. However, it has been shown for Glc that, provided the infusion protocol yields “reasonably” stable plasmatic fractional enrichment, blood sampling, as well as Michaelis–Menten kinetics, can be omitted. Cerebral Pyr/Lac fractional enrichment can be directly fitted as an additional unknown parameter (Valette etal., 2009).

### TOWARD DYNAMIC MODELING OF ISOTOPOMERS

For a given set of metabolic pathways, dynamic modeling of isotopomer time courses should, in theory, allow the derivation of metabolic fluxes with the highest achievable reliability, due to the higher information content (provided SNR is high enough). In practice, this has been performed in a very limited number of studies (e.g., Haberg etal., 1998; Serres etal., 2007), and never *invivo*. Isotopomer modeling is regularly performed *in vitro* and *exvivo* at steady-state (see Wiechert, 2001 for a review). Conversely, *in vivo* modeling in the brain has been almost exclusively performed using dynamic positional enrichments (for review of this, see de Graaf etal., 2003b; Gruetter etal., 2003; Henry etal., 2006; Rothman etal., 2011). As far as we know, isotopomer modeling has not been successfully achieved in the brain *in vivo*, although it can theoretically yield flux values with unparalleled accuracy (Shestov etal., 2012). This is probably due to the difficulty of measuring ^13^C spectra fine structure *in vivo*, especially with a high-temporal resolution to perform dynamic modeling. However, it has been shown that using high-field NMR systems allowed the dynamic detection of ^13^C isotopomers in the rat brain during an infusion of [1,6-^13^C_2_]Glc (Henry etal., 2003b) and double infusion of [1,2-^13^C_2_]acetate and [1,6-^13^C_2_]Glc (Deelchand etal., 2009). Recent modeling of these data suggests that current metabolic models are incomplete to account for the dynamics of all isotopomer time-curves (Jeffrey etal., 2013), appealing for new refined models.

### SINGLE- OR TWO-COMPARTMENT MODEL

A detailed description of models found in the literature is beyond the scope of this review. We will only briefly present the main metabolic pathways and assumptions in two popular models. The first one is the single-compartment model, which allows the measurement of TCA cycle flux (*V*_TCA_) following infusion of [1-^13^C]Glc or [1,6-^13^C_2_]Glc. The Pyr/Lac pool is in exchange with the blood pool at the rate *V*_dil_, leading to label dilution (**Figure 2B**). Measuring Glu C4 and C3 is required to derive both the *V*_TCA_ and the rate of exchange (*V*_X_) between αKG and Glu. Some early works proposed that *V*_X_ was much higher than *V*_TCA_ (Mason etal., 1992), allowing *V*_TCA_ estimation from Glu C4 only. There is still some controversy about the value of *V*_X_, and modeling of Glu C3 and C4 should be considered safer if no assumption is done on *V*_X_ (Henry etal., 2006). An Asp pool can be added to the model, in exchange with OAA at the same rate *V*_X_, to ensure nitrogen mass balance through the malate–aspartate shuttle. In this model, the Glu–Gln cycle is usually modeled by a simple exchange between both pools at a rate usually set to ~0.5×*V*_TCA_. Since most Glu is neuronal, this model essentially reflects neuronal *V*_TCA_. Extensive review of this model for *in vivo* applications was provided by Henry etal. (2003a).

An increasingly popular model (including *in vivo*) is the so-called two-compartment model, where neurons and astrocytes are explicitly considered (**Figure 2B**). Infusion of various substrates ([1-^13^C]Glc, [1,6-^13^C_2_]Glc, [2-^13^C]Glc, [1,2-^13^C_2_]acetate) may be performed to calculate simultaneously neuronal TCA cycle, glial PDH and PC fluxes, and the glutamatergic neurotransmission flux *V*_NT_ (Glu–Gln cycle). A net efflux of Gln in the blood is generally considered to remove extra carbon skeletons added by PC. Robustness of the model requires the measurement of Glu and Gln at position C4 and C3, and measurement of Asp C2 and C3 may help stabilize the model (Gruetter etal., 2001). Isotopomer modeling may significantly improve model’s reliability.

Some publications have sought to refine single-compartment or two-compartment models by including conversion of Glu to GABA and its reentry into TCA cycle, which is associated with GABAergic neurotransmission (Lapidot and Gopher, 1994; Patel etal., 2005; van Eijsden etal., 2010; Duarte and Gruetter, 2013).

### ASSESSING A MODEL’S RELIABILITY

When performing modeling, the quality and amount of measured ^13^C enrichments should be high enough for the problem to be well determined (i.e., estimated flux values should be close to the real values), and standard deviation on fluxes, as well as covariance between fluxes should be low. A method of choice to explore model’s reliability is Monte Carlo simulations. Enrichments are simulated for to-be-infused substrates and to-be-measured metabolites, using the metabolic model and given flux values. Noise is then added to yield SNR comparable to experimental SNR, and noised enrichments are fitted using the model. This procedure is repeated hundreds of times to derive mean and standard deviation for the estimated fluxes. The degree of confidence one can have in flux values can therefore be assessed for a given metabolic model and given experimental conditions. It allowed showing that estimation of *V*_TCA_ and *V*_X_ from the Glu C4 time-course only is very uncertain (Henry etal., 2006), and that the glutamatergic neurotransmission *V*_NT_ may not be reliable when only [1-^13^C]Glc or [1,6-^13^C_2_]Glc infusion is performed (Shestov etal., 2007).

## METABOLIC COOPERATION BETWEEN NEURONS AND ASTROCYTES STUDIED BY NMR SPECTROSCOPY

### ASTROCYTE–NEURON LACTATE SHUTTLE HYPOTHESIS: FOLLOWING LACTATE PRODUCTION AND CONSUMPTION BY THE BRAIN

Since the astrocytes are located between blood vessels and neurons, the question arises whether the astrocytes play the role of intermediary in the flow of substrates from blood to neurons. Indeed, Glc can reach neurons (i) directly, by diffusing from the capillaries through the intercellular space using the Glc transporters present in each of these cells (GLUT-1 and GLUT-3; Vannucci etal., 1997); or (ii) through the astrocytes, since astrocytic end-feet continuously cover blood vessel walls (Mathiisen etal., 2010). In this latter option, Glc that enters the astrocytic end-feet can be metabolized and the product can be subsequently transferred to the neurons and used as a substrate. A growing body of evidence supports this latter hypothesis and indicates that the astrocytic metabolic supply for neurons could be Lac (Dringen etal., 1993; Pellerin and Magistretti, 1994; Larrabee, 1995; Poitry-Yamate etal., 1995; Waagepetersen etal., 1998). Indeed, it has been shown that the presence of Lac in a Glc-free medium maintains synaptic activity in brain slices (Schurr etal., 1988). In addition, Lac has a protective effect and allows better recovery of neurons after hypoxia (Schurr etal., 1997). Although Lac has relatively low permeability at the BBB, different isoforms of monocarboxylate transporters have been localized on endothelial cells (MCT1; Leino etal., 1999), astrocytes (MCT1), and neurons (MCT2; Bröer etal., 1997, 1999). Moreover, the isoenzymes of LDH, LDH1 and LDH5, have been found in different cellular locations (Bittar etal., 1996), supporting the hypothesis of astrocytic Lac utilization by neurons.

**FIGURE 3:**
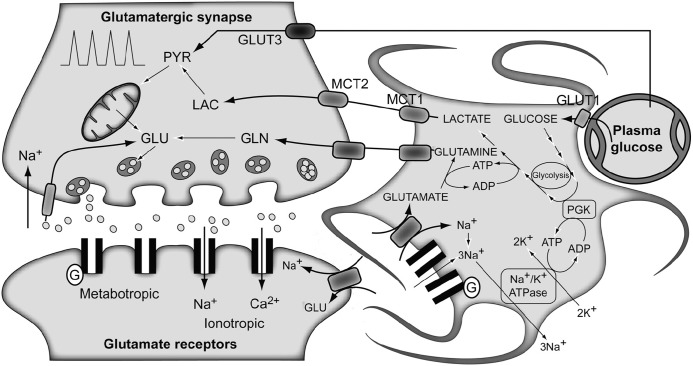
**The traditional metabolic coupling hypothesis between neurons and astrocytes during glutamatergic neurotransmission.** Glutamate released to the synaptic cleft during glutamatergic neurotransmission is co-transported with Na^+^ to the astrocytes. Astrocytic Na^+^ is exchanged by extracellular K^+^ through the Na^+^/K^+^ ATPase, consuming one ATP molecule. Astrocytic glutamate produces glutamine through glutamine synthetase, consuming one additional ATP molecule. Lactate produced exclusively in astrocytic glycolysis to support these energy demands, is extruded to the extracellular medium, taken up by the surrounding neurons and oxidized as their main metabolic fuel. Note the apparent stoichiometric coupling between glutamate–glutamine cycling and glucose uptake as well as the exclusive *glycolytic* or *oxidative* metabolisms in astrocytes and neurons, respectively. Gln, glutamine; Glu, glutamate; GLUT 1 and GLUT 3, glucose transporters 1 and 3; Lac, lactate; MCT1 and MCT2, monocarboxylate transporters 1 and 2; PGK, phosphoglycerate kinase; Pyr, pyruvate. Adapted with permission from Tsacopoulos and Magistretti (1996).

The traditional metabolic coupling theory (ANLSH for the astrocyte–neuron lactate shuttle hypothesis), firstly proposed by Pellerin and Magistretti in the mid-1990s (Pellerin and Magistretti, 1994), describes that neurotransmitter Glu released to the synaptic cleft, following an action potential, is recaptured predominantly by the high-affinity Glu transporters of surrounding astrocytes in the neuropil (**Figure 3**). Three Na^+^ atoms are co-transported with each Glu molecule to the astrocytic cytosol and metabolized into Gln by GS. The Na^+^ atoms are extruded from the cytosol through the Na^+^/K^+^ ATPase, at the expense of one ATP molecule, and GS consumes one additional ATP molecule. These two ATP molecules were originally proposed to be compensated for by the degradation of one Glc molecule in the astrocyte through the glycolytic pathway. Gln is then extruded to the extracellular space, being recaptured by the neurons to regenerate Glu. Therefore, in this particular process, we assist in the consumption of one Glc molecule from plasma, with the subsequent generation of two molecules of astrocytic Lac. These Lac molecules are exported to the neurons to become their main metabolic fuel. Thus, Gln production appears to be stoichiometrically coupled to Glc uptake (1:1 stoichiometry), glycolysis occurring mainly in the astrocytes while Pyr oxidation remaining as a predominantly neuronal process. These findings fit well with early ^13^C NMR results, which determined the cerebral Gln cycle and the TCA cycle fluxes from a minimal mathematical model. This model assumed that [4-^13^C]Glu and [4-^13^C]Gln turnover curves reflected the neuronal TCA cycle and the Gln cycle fluxes, respectively (Sibson etal., 1998). Later, Rothman etal. (2003) proposed Gln as the major precursor of cerebral Glu and the Gln cycle was found to be stoichiometrically coupled to Glc uptake, accounting for 60–80% of the energy derived from Glc metabolism.

Importantly, a thorough examination of the earlier evidences led several authors to challenge the traditional approach proposed by the ANLSH (Chih etal., 2001; Dienel and Hertz, 2001; Chih and Roberts, 2003; Dienel and Cruz, 2003). In response to these criticisms, Pellerin and Magistretti (2003) presented a revised version of their proposal. The main differences are that this newer proposal does not exclude the activation of glycolysis and production of Lac in active neurons. Additionally, it does not require a direct coupling between astrocytic Lac release and neuronal Lac oxidation, proposing that Lac from both active astrocytes and neurons is released into the extracellular space. This Lac is eventually used by neurons (at rest or during activity). The current version of the ANLSH has been also critically reviewed (Hertz, 2004).

In the context of this review, it is important to remark that both ^1^H (Prichard, 1991; Prichard etal., 1991; Merboldt etal., 1992; Sappey-Marinier etal., 1992) and ^13^C NMR spectroscopy studies have been used to explore the ANLSH/metabolic coupling theory by monitoring and comparing the fate of ^13^C-glucose and ^13^C-lactate metabolism in neurons (Sonnewald etal., 1991; Schousboe etal., 1997; Bouzier-Sore etal., 2003), astrocytes (Alves etal., 1995), rat brain (Bouzier etal., 2000; Hassel and Brathe, 2000; Serres etal., 2004; Sampol etal., 2013) and human brain (Boumezbeur etal., 2010).

### THE REDOX SWITCH/REDOX COUPLING HYPOTHESIS

Several other convincing evidences have accumulated since the above explained interpretations of metabolic neuroglial coupling during glutamatergic neurotransmission, with relevant consequences. These new pieces of evidence showed that: (i) an important portion of the energy used to synthesize Gln is derived from the astroglial TCA cycle (Garcia-Espinosa etal., 2003); (ii) up to 40% of cerebral Glu is derived from alternative sources to Gln (Garcia-Espinosa etal., 2003); (iii) Gln cycling may not present a 1:1 stoichiometry with Glc uptake (Gruetter etal., 2001; McKenna, 2007); (iv) different kinetic pools of Lac, Pyr, Gln, Glu, and GABA exist both in astrocytes and in neurons (Cruz etal., 2001; Zwingmann etal., 2001; Rodrigues etal., 2005; Cerdan etal., 2009). All the previous described findings indicate that the coupling mechanisms between neuronal and glial metabolisms are more complex than previously envisioned.

**FIGURE 4:**
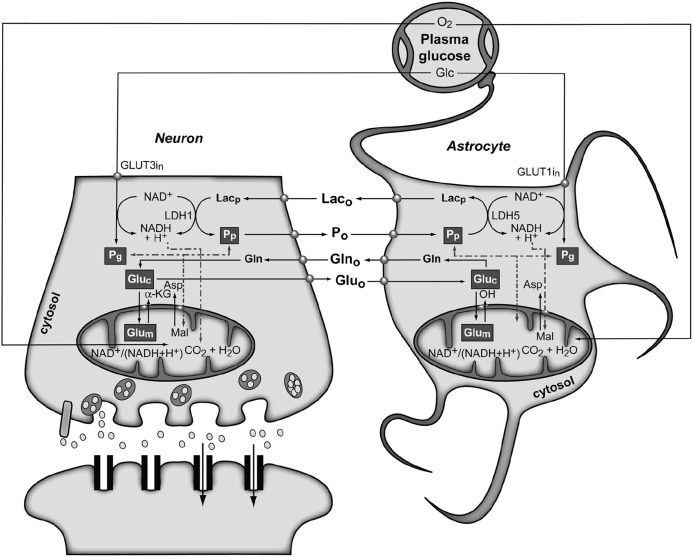
**The subcellular compartmentation of pyruvate and glutamate and the redox switch/redox coupling hypothesis.** Two pools ofpyruvate exist in neurons and astrocytes derived from extracellular monocarboxylates (Pp) or glucose (Pg). A lactate/pyruvate redox shuttle is able to transfer continuously lactate from astrocytes to neurons, taking advantage of the kinetics of plasma membrane transporters and lactate dehydrogenase isoenzymes. High cytosolic lactate concentration inhibits neuronal glycolysis at the glyceraldehyde-3-phosphate dehydrogenase step by competition with cytosolic NAD^+^, favoring the oxidation of extracellular Lac. Neuronal pyruvate is transferred back to the astrocyte to close the transcellular exchange of reducing equivalents. Two α-ketoglutarate/glutamate pools exist in neurons and astrocytes, associated probably to cytosolic and mitochondrial compartments. Exchange of α-ketoglutarate/glutamate between mitochondria and cytosol appears to be slow in the H3 glutamate hydrogen exchange timescale and dependent of the cytosolic and mitochondrial NAD(P)^+^/NAD(P)H ratios, as determined by the malate–aspartate shuttle. Both glycolysis and oxidative astrocytic metabolism contribute the energy for glutamine production in the astrocytes, indicating that this coupling involves both transcellular and intracellular redox coupling mechanisms that allow the simultaneous operation of glycolysis and oxidation in astrocytes. Asp, aspartate; Glc, glucose; Gln, glutamine; Glu, glutamate; GLUT1 and GLUT3, glutamate transporters 1 and 3; α-KG, α-ketoglutarate; Lac, lactate; LDH1 and LDH5, lactate dehydrogenase 1 and 5; Mal, malate. Reproduced with permission from Rodrigues etal. (2012).

**Figure 4** shows the conception of metabolic coupling between neurons and astrocytic during glutamatergic neurotransmission proposed by Cerdan and colleagues (Cerdan etal., 2006; Ramirez etal., 2007). This hypothesis is based on the existence of transcellular coupling of oxidative and non-oxidative metabolisms in both neurons and astrocytes through the exchange of monocarboxylate reducing equivalents and on the operation of intracellular redox switches. So, after presynaptic Glu release, astrocytes incorporate Glu and three Na^+^ ions, being the latter removed subsequently through the plasma membrane Na^+^/K^+^ ATPase. The energetic cost of this process implies reduced astrocytic ATP/ADP concentrations, stimulating astroglial glycolysis and TCA cycle. Both astrocytic metabolic pathways contribute the energy required by GS, with a major contribution of the oxidative metabolism. However, the energy demands during glutamatergic neurotransmission eventually exceed the reduced capacity of the astrocytic TCA cycle, what could result in a net activation of the glycolytic flux and a net production of astrocytic Lac, which is rapidly extruded to the extracellular space. The resulting extracellular Lac is taken up by neurons with a consequent reduction of the cytosolic redox state to a point where neuronal glycolysis could be inhibited at the glyceraldehyde 3-phosphate dehydrogenase step. An opposite flux of Pyr, from neurons to astrocytes, is proposed to connect and balance the redox state in both neurons and glial cells. Under these conditions, extracellular Lac is predominantly consumed by neuronal oxidation until its extracellular concentration reaches the pre-activation levels, preparing the stage for a new glutamatergic event.

This redox switch/redox coupling hypothesis integrates basically the described experimental findings, both obtained *in vivo* and *in vitro*. More specifically, it includes: (i) the simultaneous operation of both astrocytic and neuronal glycolysis and TCA cycles during neuronal activation; (ii) the fact that both astrocytes and neurons may potentially use Glc or Lac as complementary, or even alternative, substrates; this depends on the extracellular redox state and availability; (iii) the stoichiometric or non-stoichiometric coupling of the Glu cycle and Glc uptake; (iv) the intracellular compartmentalization of cytosolic monocarboxylates; and, finally, (v) the intracellular Glu compartmentalization also both in neurons and astrocytes (Cruz etal., 2005; Dienel and Hertz, 2005). The transcellular redox switch/redox coupling proposal mimics the intracellular coupling mechanisms existing between cytosolic glycolysis and the TCA cycle which involves the transfer of reducing equivalents through the inner mitochondrial membrane. During transcellular redox coupling, however, reducing equivalents are reversibly exchanged between neurons and astrocytes in the form of Lac and Pyr (Arco and Satrustegui, 2005; McKenna etal., 2006).

## PERSPECTIVE: HYPERPOLARIZED ^13^C NMR APPROACHES

As explained before, one of the most limiting features of NMR is its lack of sensitivity. Therefore, magnetic resonance imaging (MRI) has relied primarily on imaging of water protons. This results from the fact that the SNR ratio of the NMR signal is proportional to the equilibrium polarization between the two proton spin states under thermal equilibrium conditions in an external magnetic field (B_0_), as well as the proton concentration. Clinical imaging applications have until now been restricted to ^1^H MRI because the existence of a high concentration of protons in biological tissue is able to counterbalance the inherent low sensitivity. Unfortunately, MR sensitivity of ^13^C is too low to allow conventional ^13^C MRI due to the vestigial *in vivo* abundance of this nucleus and its lower magnetogyric ratio.

Although it is possible to improve the sensitivity using MRI systems at high B_0_ and extremely low temperatures, a maximum polarization (and corresponding SNR) increase (~10^3^), obtained by cooling down the sample to liquid He temperature at a field strength of 20T, would not be sufficient for clinical ^13^C MRI applications. Alternatively, it is possible to improve the sensitivity by transferring polarization from an electron or nuclear spin that has a higher polarization, creating a non-equilibrium distribution of nuclear spins called the hyperpolarized state (Månsson et al., 2006). In this state, the polarization of spins can be increased by a factor of ~10^5^ compared with that in the thermal equilibrium state and independently of the B_0_ value, leading to a corresponding gain in signal strength for MRI. This allows imaging of nuclei other than protons, namely ^13^C, and their molecular distribution *in vivo* can be visualized in a clinically relevant time window (Ardenkjær-Larsene et al., 2003).

The hyperpolarized state is created by an external device followed by rapid administration of the agent to the subject to be imaged. However, the lifetime of the hyperpolarized state is limited by the *T*_1_ relaxation time which depends on the chemical structure and environment of the hyperpolarized compound. In the case of ^13^C, it can range from a few seconds to several minutes, depending on the functional groups where the ^13^C nucleus is present.

Both parahydrogen-induced polarization (PHIP) and dynamic nuclear polarization (DNP) techniques have been able to hyperpolarize a wide range of organic ^13^C-labeled substances. As the polarization of electrons is much higher than the ^13^C nuclear polarization, due to the much larger gyromagnetic ratio of the electron, the DNP approach implies transferring polarization from hyperpolarized electron spins in a solid to the coupled ^13^C nuclear spins in a doping substance (~3T and ~1K; Månsson et al., 2006). Microwave irradiation near the electron resonance frequency transfers the polarization from the unpaired electrons to the ^13^C nuclei. After reaching an appropriate polarization, the solid is rapidly dissolved and injected with small polarization losses (Ardenkjær-Larsene et al., 2003).

An interesting use of ^13^C-labeled endogenous compounds is metabolic imaging. Chemical shift imaging (CSI) has been traditionally used to image the cerebral distribution of metabolites from ^1^^3^C-labeled substances, such as Glc (van der Zijden etal., 2008). However, without using hyperpolarization techniques, such images can only be obtained using long scan times (minutes). Using the previously described hyperpolarization approaches, images of the metabolic processes can be generated in a significant faster time scale (seconds). Endogenous compounds selectively labeled with ^13^C have been hyperpolarized by the DNP technique, extending substantially the applications of cerebral metabolic imaging. Basically, enzymatic processes can be non-invasively quantified and imaged *in vivo* using these hyperpolarized ^13^C-labeled metabolites. The metabolic fate of [1-^13^C]Pyr in images of tumor-bearing animals injected with hyperpolarized labeled Pyr has been followed using the DNP approach, and allowed mapping the metabolic pattern of labeled Pyr, as well as of Lac and alanine. It was confirmed that gliomas abundantly transform Pyr into Lac through anaerobic glycolysis. Using this strategy, it was shown that exchange of hyperpolarized ^13^C label between Pyr and Lac could be imaged in tumors (Day etal., 2011). This flux was decreased in tumors receiving treatment undergoing drug-induced cell death. Using the same substrate, fast dynamic spiral CSI and transport modeling were combined to better characterize the bolus, transport, and metabolic effects, separating the metabolites in the cerebral blood volume from the metabolites in the brain tissue. This allowed developing a repeatable non-invasive measurement of regional BBB transport kinetics and regional cerebral Lac levels (Hurd etal., 2010). A novel non-invasive method for imaging tissue pH *in vivo* was also demonstrated (Gallagher etal., 2008). It was shown that interstitial tumor pH can be imaged *in vivo* from the ratio of the signal intensities of hyperpolarized bicarbonate (H^13^CO_3_^-^) and ^13^CO_2_, after the intravenous injection of hyperpolarized H^13^CO_3_^-^. Additionally, other neurochemical pathways have been exploited using this approach. Conversion of ^13^C-labeled acetate to 2-oxoglutarate, a key biomolecule connecting metabolism to neuronal activity, was recently shown using the DNP approach, reporting a direct *in vivo* observation of a TCA cycle intermediate in intact brain (Mishkovsky etal., 2012). The cerebral distribution and metabolism of hyperpolarized 2-keto[1-^13^C]isocaproate (KIC) has also been described in the normal rat using MR (Butt etal., 2012). Hyperpolarized KIC is metabolized to [1-^13^C]leucine by branched chain amino acid transaminase, having this enzyme an important role in nitrogen shuttling and glutamate metabolism in the brain. Another group was able to show how sodium 1-^13^C acetylenedicarboxylate, which after hydrogenation by PASADENA (Parahydrogen and Synthesis Allows Dramatically Enhanced Nuclear Alignment), becomes ^13^C sodium succinate. Fast *in vivo* imaging demonstrated that, following carotid arterial injection, the hyperpolarized^13^C-succinate appeared in the head and cerebral circulation of normal and tumor-bearing rats (Bhattacharya etal., 2007). Even more recently, the injection of hyperpolarized [U-^2^H,U-^13^C]Glc allowed real-time imaging of the glycolytic flux in two non-cerebral murine tumor models *in vivo*, due to the clear detection of labeled Lac (Rodrigues etal., in press). Low levels of dihydroxyacetone phosphate, 6-phosphogluconate and bicarbonate were also observed, with the latter two synthesized by the pentose phosphate pathway activity. The possible use of labeled Glc in cerebral studies could open a very important avenue in neurochemistry, mainly because of the possibility to investigate a completely new metabolic timeframe with this approach. Therefore, the application of ^13^C metabolic imaging using hyperpolarized ^13^C-labeled substrates to neurochemistry is an open field of research.

## Conflict of Interest Statement

The authors declare that the research was conducted in the absence of any commercial or financial relationships that could be construed as a potential conflict of interest.

## Abbreviations

AcCoA: acetyl-CoA
αKG: α-ketoglutarate
ANLS: astrocyte–neuron lactate shuttle
Asp: aspartate
ATP: adenosine triphosphate
B_0_: external magnetic field
BBB: blood–brain barrier
Cho: choline
CMR_glc_: cerebral metabolic rates for glucose
CNS: central nervous system
CP: cross-polarization
Cr: creatine
CSI: chemical shift imaging
DNP: dynamic nuclear polarization
FDG: 2-(^18^F)-deoxyglucose-6-phosphate
fMRI: functional magnetic resonance imaging
GABA: γ–ã-aminobutyric acid
Glc: glucose
Gln: glutamine
Glu: glutamate
GLUT: glucose transporter
GS: glutamine synthetase
Ins: *myo*-inositol
KIC: 2-ketoisocaproate
Lac: lactate
LDH: lactate dehydrogenase
Mal: malate
MCT: MCT, monocarboxylate transporter
MRI: magnetic resonance imaging
NAA: *N-acetyl-aspartic acid*
NMR: nuclear magnetic resonance
nOe: nuclear Overhauser effect
OAA: oxaloacetate
OAAn: neuronal OAA
PC: pyruvate carboxylase
PCr: phosphocreatine
PDE: phosphodiesters
PDH: pyruvate dehydrogenase
PET: positron emission tomography
PGK: phosphoglycerate kinase
PHIP: parahydrogen-induced polarization
Pi: norganic phosphate
PME: phosphomonoesters
POCE: proton-observed carbonedited
PPT: pulsed polarization transfer
Pyr: pyruvate
RF: radiofrequency
SNR: signal-to-noise ratio
Suc: succinate
TCA: tricarboxylic acid
VNT: glutamatergic neurotransmission flux
VTCA: TCA cycle flux
VX: rate of exchange between α-ketoglutarate and glutamate.
